# Research Progress on the Correlation Between *Gardnerella* Typing and Bacterial Vaginosis

**DOI:** 10.3389/fcimb.2022.858155

**Published:** 2022-03-25

**Authors:** Hanyu Qin, Bingbing Xiao

**Affiliations:** Department of Obstetrics and Gynecology, Peking University First Hospital, Beijing, China

**Keywords:** *Gardnerella*, bacterial vaginosis, clinical outcomes, subtypes, qPCR, cpn-60, whole genome sequencing

## Abstract

Bacterial vaginosis (BV) is the most common infectious disease of the reproductive tract in women of childbearing age. It often manifests as an imbalance in the vaginal microbiome, including a decrease in *Lactobacillus* and an increase in anaerobic bacteria. While *Gardnerella* spp. are considered a major cause of BV, they are also detected in the vaginal microbiome of healthy women. *G. vaginalis* was the only recognized species of *Gardnerella* until a recent study characterized three new species, *G. leopoldii*, *G. piotii*, and *G. swidsinskii*. This review describes the different types and genetic diversity of *Gardnerella*, as well as new findings on the correlation between different *Gardnerella* spp. and BV.

## Introduction

Bacterial vaginosis (BV) is the most common lower genital tract infection affecting approximately 30% of women in the general population and 50% of African American women (Ravel et al., 2011; Kenyon et al., 2013; Morrill et al., 2020). Variations in prevalence exist among different races and ethnicities (Allsworth and Peipert, 2007). BV is associated with a disruption of the optimal vaginal microbiota characterized by a decreased proportion of lactic acid-producing bacteria and an increased proportion of a wide array of strict and facultative anaerobes (Ravel et al., 2021). Bacteria commonly associated with BV include *Gardnerella vaginalis* (*G. vaginalis*), *Megasphaera* spp., *Fannyhessea vaginae* [previously known as *Atopobium vaginae*, (Rodriguez Jovita et al., 1999; Nouioui et al., 2018)], *Dialister* spp., *Mobiluncus* spp., *Sneathia amnii*, *Sneathia sanguinegens*, *Porphyromonas* spp., and Prevotella spp (Muzny et al., 2018; Rosca et al., 2020). Although BV is frequently asymptomatic, women with BV are more likely to report vaginal odor, itching, and discharge than those without (Klebanoff et al., 2004). In addition, most women do not report BV symptoms to their providers, even when there are clinical signs (Masson et al., 2019). Serious adverse health outcomes have been associated with BV, including increased risk of infertility (Ravel et al., 2021), adverse pregnancy outcomes (Giakoumelou et al., 2016; Tabatabaei et al., 2019), pelvic inflammatory disease (PID) (Wiesenfeld et al., 2002; Ravel et al., 2021) and sexually transmitted infections (STIs), including chlamydia (Shipitsyna et al., 2020), gonorrhea (Bautista et al., 2017), human papilloma virus (HPV) (Usyk et al., 2022) and human immunodeficiency virus (HIV) (Atashili et al., 2008).

BV etiology is controversial and not yet fully understood. According to Catlin’research, the G. vaginalis-associated vaginal syndrome was earlier called nonspecific vaginitis (NSV) in recognition of the absence of recognized agents of vaginitis (Catlin, 1992). In 1955, Gardner and Dukes showed that 90% of NSV cases were caused by a single microbe, *Haemophilus vaginalis* (*H. vaginalis*), and the name was changed to *H. vaginalis* vaginitis (Gardner and Dukes, 1955). However, subsequent studies found that because *H. vaginalis* does not require heme or nicotinamide adenine dinucleotide for growth, it may not be a member of the *Haemophilus* genus. *H. vaginalis* shows uncertainty in gram staining, also a characteristic of the *Corynebacteria*. As a result, *H. vaginalis* was reassigned to the *Corynebacterium* genus and renamed *Corynebacterium vaginale* (Zinnemann and Turner, 1963). This name also proved to be inaccurate because these bacteria are catalase-negative and do not have arabinose in their cell wall (Catlin, 1992). In 1980, two large taxonomic studies using DNA hybridization, electron microscopy, and various biochemical methods showed that the bacterium lacked close similarity to any previously established genus (Greenwood and Pickett, 1980; Piot et al., 1980). This resulted in the development of a new genus, *Gardnerella*, and *Corynebacterium* vaginitis was renamed *Gardnerella* vaginitis. Gardner believed that *Gardnerella* vaginitis was a specific vaginal infection with a clear cause and that most vaginitis previously classified as “nonspecific” was likely caused by *Gardnerella* spp. In 1984, the name was officially changed to “BV” because vaginitis is suggestive of an inflammatory response in the vaginal epithelium, which is usually absent in women with BV (Catlin, 1992). It wasn’t until 1984 that BV was officially named (Workshop on Anaerobic Curved Rods and Bacterial Vaginosis, Stockholm, January, 1984) (Bump et al., 1984).

Since *Gardnerella* spp. is highly detected in BV, it appears to have a special role in vaginal microbiota dysbiosis (Reid, 2018). While *Gardnerella* spp. is found in 95% to 100% of BV cases (Muzny et al., 2019), colonization does not always lead to BV (Hickey and Forney, 2014; Machado et al., 2015). Indeed, the role of *Gardnerella* spp. in BV has remained controversial because it is present in both healthy vaginal microbiota and in BV (Zozaya-Hinchliffe et al., 2010). As a result, researchers have speculated about whether there are different *Gardnerella* spp. (Cornejo et al., 2018) with distinct pathogenicities that can lead to different clinical outcomes (Janulaitiene et al., 2018). It is possible that while healthy women are colonized by a less virulent strain of *Gardnerella* spp., other more virulent strains promote the development of BV. Studies have used several *Gardnerella* spp. typing tests to explore the clinical characteristics of different species. Indeed, the taxonomy of *Gardnerella* spp. will need to be more completely defined in order to fully understand the mechanism of *Gardnerella* spp. in BV pathogenesis (Castro et al., 2019). This study reviews recent literature to explore the characteristics of *Gardnerella* spp., the typing methods used, and their clinical significance.

## Characteristics of *Gardnerella*

*G. vaginalis* was the first recognized *Gardnerella* species (Harwich et al., 2010), with its closest relatives in the Bifidobacterium genus (Harwich et al., 2010; Castro et al., 2019). The cells are small, nonmotile, nonencapsulated, non-spore-forming, pleomorphic rods with average dimensions of 0.4×1.0~1.5µm (Onderdonk et al., 2016). The cells are small, nonmotile, nonencapsulated, non-spore-forming, pleomorphic rods with average dimensions of 0.4×1.0~1.5µm (Sadhu et al., 1989). Known as a facultative anaerobic microorganism, *G. vaginalis* was also described as fastidious, as it grew better at 37°C in complex media in an atmosphere with 5–10% of carbon dioxide (CO2) or in a candleflame extinction jar (Catlin, 1992; Cereija et al., 2013). Nevertheless, it was demonstrated that certain *G. vaginalis* strains are strict anaerobes (Malone et al., 1975). The cellular surface of *G. vaginalis* is covered with fimbriae, which are responsible for the attachment of *G. vaginalis* to vaginal epithelial cells (Scott et al., 1989; Onderdonk et al., 2016). More recent biochemical testing has shown that *G. vaginalis* is catalase-, oxidase-, and b-glucosidase-negative (Catlin, 1992; Turovskiy et al., 2011). It can ferment starch, dextrin, sucrose, glucose, fructose, ribose, maltose and raffinose. Some strains can also ferment xylose and trehalose. Conversely, *G. vaginalis* is unable to ferment rhamnose, melibiose, mannitol and sorbitol (Harwich et al., 2010).

The important pathogenic characteristics of *Gardnerella* spp. include the production of sialidase, an enzyme that degrades cervical and vaginal mucus, and vaginolysin, a lysoid that induces the lysis of vaginal epithelial cells (Castro et al., 2019). Sialidase is associated with the degradation of key mucosal protective factors, such as mucin, that lead to the shedding of vaginal epithelial cells (Lewis et al., 2013; Hardy et al., 2017). *Gardnerella* spp. has three genes that encode sialidase, NanH1 (sialidase A gene), NanH2, and NanH3. NanH2 and NanH3 appear to be the main sources of sialidase activity in *Gardnerella* spp (Robinson et al., 2019). Vaginal hemolysin is a cholesterol-dependent cell hemolysin that was first discovered in the culture medium of *Gardnerella* spp. It has cytotoxic activity against human erythrocytes and can induce the dissolution of human erythrocytes, epithelial cells, and polymorphonuclear leukocytes (Zilnyte et al., 2015). Vaginal hemolysin and sialidase are the most widely studied virulence factors of *Gardnerella* spp. (Santiago et al., 2011; Janulaitiene et al., 2018), and are assumed to play a substantial role in the pathogenesis of BV (Pleckaityte et al., 2012). Swidsinski et al. used fluorescence *in situ* hybridization (FISH) specific for *Gardnerella* spp. and were the first to show that these species form biofilms on vaginal epithelium in women with BV. This finding revealed the nature of clue cells, epithelial cells covered by a *Gardnerella* spp. biofilm, and their etiological role in *Gardnerella* spp. infection (Swidsinski et al., 2005).

## *Gardnerella* spp. Typing

### Biotyping

*Gardnerella* spp. was first divided into eight biotypes based on its metabolic characteristics (Piot et al., 1984). In 1894, Piot et al. established a simple and repeatable method for *Gardnerella* spp. biotyping, known as Pilot typing, that is based on the reaction of lipase, hippurate hydrolysis, and β-galactosidase. Benito et al. identified 17 *Gardnerella* spp. biotypes based on these characteristics in addition to fermentation of xylose, arabinose, and galactose (Benito et al., 1986). This method is based on enzyme reactions, however, so there is a risk that the wrong biotypes will be produced when conditions change. Thus, biotyping is not recommended to understand the epidemic etiology of *Gardnerella* spp. (Ingianni et al., 1997).

### Genotyping

#### Amplified Ribosomal DNA Restriction Analysis

Four genotypes of *Gardnerella* spp. were identified by amplified ribosomal DNA restriction analysis (ARDRA) using different restriction enzymes (Ingianni et al., 1997). In 1997, Ingianni et al. first used ARDRA to identify 34 *Gardnerella* spp. strains. This method is more accurate than biotyping but still relies on the isolation and purification of *Gardnerella* spp. Each subtype has a distinct growth and metabolic pattern, however, and preferentially isolated strains may misrepresent the clinical distribution of *Gardnerella* subtypes.

#### qPCR

Quantitative polymerase chain reaction (qPCR) typing can be used on uncultured clinical specimens, allowing for quantitative assessment of bacterial load and qualitative identification of *Gardnerella* subtypes (Balashov et al., 2014). Since the isolation of living bacterial cells is not involved, this approach can be performed on different types of samples, including frozen DNA or vaginal swab samples collected and stored under sub-optimal conditions. In 2012, Ahmed et al. conducted a comparative genomic analysis of 17 *Gardnerella* spp. clinical isolates and suggested that it would be more accurate to define *Gardnerella* spp. as four separate species (Ahmed et al., 2012). Balashov and his colleagues developed multiplex single-tube qPCR targeting genes encoding putative a-L-fucosidase (clade 1), a hypothetical protein (clade 2), thioredoxin (clade 3) and CIC family chloride transporter (clade 4), which was proven to be clade-specific and capable of strain typing and identification of the four *G. vaginalis* clades in noncultured clinical vaginal specimens (Balashov et al., 2014).

#### Cpn-60 Sequencing

Chaperonin-60 is a molecular chaperone required for the folding and assembly of proteins and protein complexes in all eubacteria and in the plastids and mitochondria of eukaryotes (Hill et al., 2005). A 549-567 bp fragment of the cpn-60 gene was amplified by universal PCR primers, and the sequencing results were relatively stable (Jayaprakash et al., 2012). *Gardnerella* spp. comprises four subgroups A, B, C, and D, based on cpn60 barcode sequences and whole-genome sequences (Jayaprakash et al., 2012; Schellenberg et al., 2016). Cpn60 sequencing was also applied to vaginal microbes in a larger sample of African commercial sex workers (Schellenberg et al., 2011). Profiling of vaginal microbiomes using cpn60 barcode sequencing, and application of clade-specific PCR showed that the vagina is often colonized by multiple *Gardnerella* spp. subgroups simultaneously (Khan et al., 2019).

#### Whole Genome Sequencing

Whole genome sequencing refers to the analysis of the whole genome sequence of *Gardnerella* using digital DNA-DNA hybridization and average nucleotide identity. In 2019, Vaneechoutte et al. analyzed the whole genome sequence of 81 *Gardnerella* spp. and confirmed the existence of 13 subgroups, GSP01-GSP13, all of which are sufficiently different to be classified as independent species. Two subgroups were found in subtypes clade 1, clade 2, and clade 4, and three undefined subgroups were found in subtype clade 3 (Vaneechoutte et al., 2019). This was an important breakthrough in this field that resulted in an amended description of *G. vaginalis* and the characterization of three additional *Gardnerella* species, *G. leopoldii, G. piotii*, and *G. swidsinskii* (Potter et al., 2019).

### The Relationship Between Different *Gardnerella* Subtypes

The commonly used *Gardnerella* typing methods include ARDRA, cpn-60, qPCR, and whole genome sequencing (Castro et al., 2019). Since ARDRA typing of *Gardnerella* results does not specifically correspond to clinical status (Piot et al., 1984; Benito et al., 1986), it is not detailed here. Cpn-60 gene sequencing was used to divide *Gardnerella* into four subtypes A, B, C and D, based on the phylogenetic relationship between each cpn60 gene subsequence (Jayaprakash et al., 2012). Similarly, qPCR divided *Gardnerella* isolates into four clades 1, 2, 3 and 4 (Balashov et al., 2014). Schellenberg et al. compared cpn60 UT sequences from 112 *G. vaginalis* isolates from three continents with cpn60 UT sequences extracted from previously published whole genome sequences. All new isolates and previously published genomes fall into four cpn60 subgroups (Jayaprakash et al., 2012), including 17 isolates belonging to clades 1- 4 (Ahmed et al., 2012). These results indicate that similar phylogenetic resolution can be achieved using a partial single gene sequence (552 bp) as 473 full-length gene sequences common to all 17 genomes. Cpn60 subgroups A, B, C, and D correspond to clade 4, 2, 1, and 3, respectively (Schellenberg et al., 2016). Using a retrospective comparative analysis of 103 publicly available genomes and meta-transcriptomic bacterial vaginosis datasets, Potter et al. identified 9 genome species of *Gardnerella*, GS01-09 (Potter et al., 2019). *Gardnerella* species, labeled with names or numbers, were defined by analysis of 81 genome sequences by digital DNA-DNA hybridization, average nucleotide identity (ANI), and MALDI-MS protein signatures (Vaneechoutte et al., 2019). The specific corresponding relationship is shown in **Table 1**.

**Table 1:** The relationship between different *Gardnerella* species.

Cpn-60	qPCR	*Gardnerella* genomospecies	*Gardnerella* species
Subgroup A	Clade 4	GS03	*G. swidsinskii*, *G. leopoldii*
Subgroup B	Clade 2	GS02 & GS06	*G. piotii*, Gsp03
Subgroup C	Clade 1	GS01	*G. vaginalis* (Gsp01), Gsp02
Subgroup D	Clade 3	GS05	Gsp08, Gsp09, & Gsp10
		GS04	Gsp07
		GS06	Gsp11
		GS07	Gsp12
		GS08	Gsp13
		GS09	

Four subgroups of Gardnerella have been defined by sequencing polymorphisms within the cpn60 gene and by qPCR detection of clade-specific genes (clades 1, 2, 3 and 4). These subtypes were designated as genovars by genome sequencing of 17 isolates. Genomospecies (GS) were defined by integrating four sequence comparison methods of 103 genomic sequences. Gardnerella species, labelled with names or numbers, were defined by analysis of 81 genome sequences by digital DNA-DNA hybridization, average nucleotide identity (ANI) and MALDI-MS protein signatures.

## Correlation Between Bacterial Vaginosis and *Gardnerella* Subtypes

### *Gardnerella* and Bacterial Vaginosis

Gardnerella is detected in both the vaginal secretions of women with BV and healthy women. Using two qPCR assays, Balashov et al. analyzed *G. vaginalis* bacterial loads and clade distribution in 100 clinical vaginal-swab samples and showed that the prevalence of *G. vaginalis* was 100% of BV patients and 97% in healthy women; however, the *G. vaginalis* concentration was significantly lower in non-BV samples. The detection frequency of clades 1, 2, 3 and 4 was 53%, 25%, 32% and 83%, respectively. In addition, 70% of BV vaginal swab samples had multiple subtypes of *Gardnerella* (Balashov et al., 2014). An assessment of the vaginal microbiota of 413 non-pregnant, reproductive-age Canadian women showed that the number of *Gardnerella* spp. detected per *Gardnerella*-positive sample ranged from 1 to 10. Multiple *Gardnerella* spp. were detected in 63.8% of samples, consistent with a previous report of multiple *Gardnerella* clades in 70% of samples from women (Balashov et al., 2014), and the majority contained one or two species (Hill et al., 2019). Multiple subtypes of *Gardnerella* had a significant positive correlation with BV, suggesting that the co-occurrence of multiple subtypes may be one of the risk factors for BV.

### Clinical Symptoms Associated With *Gardnerella* Subtypes

There was no correlation between *Gardnerella* spp. biotyping and clinical symptoms (Pleckaityte et al., 2012). According to Hill and Albert’s research, the relative abundances of the more frequently occurring species (*G. vaginalis*, *G. swidsinskii*, *G. leopoldii*, *G. piotii*, and genome species) among groups based on clinical Nugent scores (negative, intermediate, and BV) and self-reported symptoms in the 2 weeks prior to the swab collection (odor, irritation, and discharge) were compared (Hill and Albert, 2019). Whole genome sequence analysis revealed a strong relationship between abnormal odor and discharge with higher relative abundance of *G. vaginalis* and *G. swidsinskii*. The cooccurrence of these *Gardnerella* spp. also showed proportionality, suggesting that their abundance is correlated. Thus, it remains unclear whether one of these species or both are associated with vaginal symptoms (Hill and Albert, 2019).

### *Gardnerella* Subtypes Distribution

Early studies on *Gardnerella* typing indicated that biotypes 1, 2, and 5 were more common in females with BV (Piot et al., 1984), however subsequent studies could not confirm these findings. Balashov et al. found a positive correlation between BV and clade 1 and clade 3 in vaginal samples from 60 American women. Meanwhile, clade 2 was positively correlated with vaginal microbiota in an intermediate state of BV and C4 had no correlation with infection (Balashov et al., 2014). Janulaitiene et al. performed qPCR on vaginal swab samples from 109 Lithuanian women and confirmed that the microbial status of the vaginal microbiota was associated with the clade 1 and clade 2 subgroups (Nugent score 7-10). The clade 3 and clade 4 showed no association with high Nugent Scores (Janulaitiene et al., 2017). However, the results from a study on the vaginal microbiota of 299 Russian women of reproductive age were different from those of previous studies. Quantifying the four *Gardnerella* subtypes could more accurately distinguish BV from healthy microbiota than detecting the sialidase A gene and clade 4 was closely related to the status of the BV microbiome (Shipitsyna et al., 2019).

Plummer et al. studied the relationship between infection with the clade 1, clade 2, and clade 3 subtypes and Nugent scores in 101 Australian women of reproductive age. Multiple *Gardnerella* subtypes and the clade 1 subtype alone were associated with the absence of *Lactobacillus* in the vaginal microbiome. Clade 4 was not associated with BV or the absence of *Lactobacillus*, supporting the existence of symbiotic and pathogenic subtypes of *Gardnerella* spp (Plummer et al., 2020). Hill et al. used whole genome sequencing to compare the species abundance of *Gardnerella* subgroups between healthy women and those with BV. The relative abundance of *G. vaginalis, G. swidsinskii*, and *G. piotii* correlated significantly with Nugent scores. In addition, the association between *G. piotii* (B subgroup/clade 2) and the “intermediate state” microbiome was observed by cpn60 sequencing and qPCR (Hill and Albert, 2019). *Gardnerella* typing results from different studies are shown in **Table 2**.

**Table 2:** Different *Gardnerella* subtypes predict distinct BV clinical outcomes.

	BV	BV Intermediate	Healthy	Methods
Jayaprakash et al., 2012	Subgroup B	–	–	Cpn-60
Balashov et al., 2014	Clades 1 and 3	Clade 2	Clade 4	qPCR
Janulaitiene et al., 2017	Clades 1 and 2	–	–	clade-specific PCR
Shipitsyna et al., 2019	Clade 4	–	–	clade-specific PCR
Plummer et al., 2020	Clades1, 2, 3 and multiple subtypes	Clade 3	Clade 4	qPCR
Hill and Albert, 2019	*G. vaginalis, G. swidsinskii*, and *G. piotii*	–	–	Whole Genome Sequencing

In summary, current studies remain unable to determine the relationship between different *Gardnerella* genotypes and clinically relevant BV status.

### *Gardnerella* Subtypes Drug Resistance

Metronidazole resistance by *Gardnerella* isolates is likely responsible for refractory or recurrent BV. Women with BV are typically infected with multiple *Gardnerella* spp. subtypes, so metronidazole may eliminate sensitive *Gardnerella* subtypes but allow drug-resistant subtypes to survive. This could explain the presence of *Gardnerella* in the vaginal microbiota even after metronidazole treatment. BV is characterized by a thick vaginal multi-species biofilm, in which *Gardnerella* spp. is the predominant species. Since standard antibiotics, like metronidazole, are unable to fully eradicate the vaginal biofilm, this may explain the high recurrence rates of BV (Machado et al., 2015; Verstraelen and Swidsinski, 2019). Early identification of metronidazole-resistant *Gardnerella* subtypes helps to predict the prognosis of BV and inform an appropriate treatment plan. To explore the sensitivity of different *Gardnerella* subtypes to metronidazole, Schuyler et al. collected 88 *Gardnerella* strains and divided them into four groups, clades 1, 2, 3 and 4, by qPCR sequencing. Metronidazole resistance was defined as a minimum inhibitory concentration ≥32 μg/mL. A high correlation was found between metronidazole resistance and *Gardnerella* typing. While clade 3 and clade 4 strains showed 100% resistance, while clade 1 and clade 2 showed 35% and 7.1% resistance, respectively (Schuyler et al., 2016).

### Clinical Outcomes of *Gardnerella* Subtypes

In 2017, Hilbert et al. conducted a short-term longitudinal study of 149 non-pregnant Canadian women of reproductive age. Vaginal swab samples were collected at first diagnosis, 7 days after treatment, and 40-45 days after follow-up to detect the abundance of different *Gardnerella* subtypes by qPCR. Higher prevalence of clade 1 and clade 4 were found in vaginal samples than clade 2 and clade 3. The abundance of each subtype increased as the Nugent score, or the severity of BV, worsened (Hilbert et al., 2017). The abundance of clade 1 and clade 4 decreased after clinical treatment regardless of the clinical and microbiological outcome, and clade 2 decreased in women who continued treatment for 40-45 days. Recurrent BV is characterized by increased clade 1 and clade 2 levels after treatment. The clade 1 and clade 4 subgroups were the dominant strains in vaginal specimens. While the clade abundance of *Gardnerella* was generally higher in vaginal samples that met the four Amsel criteria than those that did not, clade 1 was an exception. Thus, differences in qPCR subtype abundance were associated with Nugent score, Amsel criteria, sensitivity to treatment, and BV recurrence.

A recent study by Turner et al. associated sustained high abundance of the *Gardnerella* Gsp07 subtype with a refractory BV response and sustained low abundance of the *Gardnerella* Gsp07 subtype and *G. Swidsinskii/G. Leopoldii* with BV remission. In most patients with BV relapse or remission, the abundance of *Lactobacillus* species increased 4–14 days after initiation of treatment, and the increase was more obvious and sustained in patients with BV remission. These findings confirmed that Gardner’s Gsp07 subtypes and *G. swidsinskii/G. leopoldii* coinfection correlate with poor clinical outcomes. Alternatively, direct or indirect inhibition of lactic acid bacteria strains may interfere with clinical recovery. Treatment by clinicians targeting these marker subtypes of adverse outcomes may improve clinical outcomes in patients with BV (Turner et al., 2021).

### Virulence Factors of *Gardnerella* Subtypes

*Gardnerella* pathogenicity is primarily mediated through vaginal hemolysin (VLY), sialidase and biofilm formation (Pleckaityte et al., 2012). Previous studies have shown that pathogenicity differs by *Gardnerella* subspecies (Janulaitiene et al., 2018). Zilnyte et al. found that VLY activity is dependent on the complement regulatory molecule, CD59, and showed that higher CD59 expression in hamsters correlated with increased vaginal hemolysin-soluble cell sensitivity (Zilnyte et al., 2015). In the cell culture model, the expression level of vaginal hemolysin was correlated with the level of cytotoxicity, but there was no any correlation between VLY production level and *G. vagina*lis genotype/biotype (Pleckaityte et al., 2012).

Sialidase lyses the terminal sialic acid residues of sialoglycan in the vaginal environment and plays a key role in providing nutrition for *Gardnerella* spp. through sialic acid catabolism, providing a site for bacteria adhesion to the epithelium, facilitating biofilm formation, and modulating immune responses (Lewis et al., 2013; Schellenberg et al., 2017). Harwich et al. (2010) and Janulaitiene et al. (2018) found significant differences in the sialidase activity of *Gardnerella* clades, however, with clade 2 having the highest levels followed by clade 1, and clade 4 having the lowest. In addition, the gene coding for sialidase was detected in all isolates of clade 1 and clade 2, but not in clade 4 isolates (Schellenberg et al., 2016; Janulaitiene et al., 2017). Shipitsyna also holds that clade 4 strains mostly lack the sialidase A gene (Shipitsyna et al., 2019). Sialidase activity is considered a marker of BV. Indeed, more than 50% of BV is asymptomatic, which may be caused by *Gardnerella* subspecies that lack sialidase (Janulaitiene et al., 2017). Sialidase acts on sugar chains with sialic acid residues, which are abundant on the mucosal surface of the reproductive tract. Sialidase activity can be used as a diagnostic marker of BV (Janulaitiene et al., 2018) and rapid clinical detection using products like BVBlue^®^ (Sekisui Diagnostics, L.L.C., Birmingham, AL, USA) (Hill and Albert, 2019).

The exfoliated vaginal epithelial cells in BV patients are covered with multi-bacterial biofilms dominated by *Gardnerella* (Vestby et al., 2020). Biofilm formation is not only associated with increased antimicrobial resistance and disease recurrence but also increased risk of sexual transmission. However, there is no significant difference in biofilm quantity between *Gardnerella* subtypes (Janulaitiene et al., 2018).

## Conclusions

In summary the relationship between the different subtypes of *Gardnerella* and bacterial vaginosis is unclear. A large number of studies show that different *Gardnerella* subtypes are possibly represent different drug resistance, virulence, bacterial load and indicate the clinical outcomes of BV. And the clinical significance of asymptomatic BV remains unclear, one possible explanation for its occurrence is the presence of high numbers of nonpathogenic *Gardnerella* or other morphologically similar species. This is especially likely given that *Gardnerella* is one of the key predictors of the Nugent score. Furthermore, metronidazole treatment for BV cure rate is not ideal, and the proportion of refractory and recurrent BV continues to rise. This study reviewed the relationship between *Gardnerella* subtypes species and BV clinical outcomes and evaluated patient prognosis according to *Gardnerella* typing. This is particularly important so that appropriate treatment can be given to improve the BV clinical cure rate and reduce adverse obstetric and gynecological complications as well as disease recurrence. Given the current diversity of *Gardnerella* phenotypes, especially virulence factors, genotypic diversity, and *Gardnerella* prevalence in women, understanding the clinical significance of these different strains is critical.

## Author Contributions

HQ and BX discussed the contents, wrote, reviewed, and edited the manuscript. All authors contributed to the article and approved the submitted version.

## Funding

This present work was funded by the grants of the National Key Research and Development Program of China (2021YFC2301000) and the National Natural Science Foundation of China (81971342).

## Conflict of Interest

The authors declare that the research was conducted in the absence of any commercial or financial relationships that could be construed as a potential conflict of interest.

## Publisher’s Note

All claims expressed in this article are solely those of the authors and do not necessarily represent those of their affiliated organizations, or those of the publisher, the editors and the reviewers. Any product that may be evaluated in this article, or claim that may be made by its manufacturer, is not guaranteed or endorsed by the publisher.
